# Murine Cytomegalovirus Infection of Neural Stem Cells Alters Neurogenesis in the Developing Brain

**DOI:** 10.1371/journal.pone.0016211

**Published:** 2011-01-13

**Authors:** Manohar B. Mutnal, Maxim C-J. Cheeran, Shuxian Hu, James R. Lokensgard

**Affiliations:** 1 Neuroimmunology Laboratory, Department of Medicine, Center for Infectious Diseases and Microbiology Translational Research, University of Minnesota, Minneapolis, Minnesota, United States of America; 2 Department of Veterinary Population Medicine, University of Minnesota, Minneapolis, Minnesota, United States of America; Cedars-Sinai Medical Center and University of California Los Angeles, United States of America

## Abstract

**Background:**

Congenital cytomegalovirus (CMV) brain infection causes serious neuro-developmental sequelae including: mental retardation, cerebral palsy, and sensorineural hearing loss. But, the mechanisms of injury and pathogenesis to the fetal brain are not completely understood. The present study addresses potential pathogenic mechanisms by which this virus injures the CNS using a neonatal mouse model that mirrors congenital brain infection. This investigation focused on, analysis of cell types infected with mouse cytomegalovirus (MCMV) and the pattern of injury to the developing brain.

**Methodology/Principal Findings:**

We used our MCMV infection model and a multi-color flow cytometry approach to quantify the effect of viral infection on the developing brain, identifying specific target cells and the consequent effect on neurogenesis. In this study, we show that neural stem cells (NSCs) and neuronal precursor cells are the principal target cells for MCMV in the developing brain. In addition, viral infection was demonstrated to cause a loss of NSCs expressing CD133 and nestin. We also showed that infection of neonates leads to subsequent abnormal brain development as indicated by loss of CD24(hi) cells that incorporated BrdU. This neonatal brain infection was also associated with altered expression of Oct4, a multipotency marker; as well as down regulation of the neurotrophins BDNF and NT3, which are essential to regulate the birth and differentiation of neurons during normal brain development. Finally, we report decreased expression of doublecortin, a marker to identify young neurons, following viral brain infection.

**Conclusions:**

MCMV brain infection of newborn mice causes significant loss of NSCs, decreased proliferation of neuronal precursor cells, and marked loss of young neurons.

## Introduction

Cytomegalovirus (CMV) is the most common infectious cause of developmental disorders of the central nervous system (CNS) in humans and the predominant cause of developmental neurological disabilities in the United States [1]. Each year, approximately 1% of all newborns have congenital CMV infection. Approximately 5 to 10% of these infected infants manifest signs of serious neurological defects at birth, including deafness, mental retardation, blindness, microencephaly, hydrocephalus, and cerebral calcification [2], [3], [4]. Thus, it seems likely that CMV infection of the fetus alters the “normal blueprint” of the developing brain, resulting in long-term neurological sequelae.

Using a murine infection model, we have previously shown that NSCs in the adult brain appear to be the predominant cell type affected by murine cytomegalovirus (MCMV) [5]. There is an abundance of NSCs in the fetal brain, in this study we will use the term neural stem cells to refer to all classes of immature and proliferating cells that reacted with CD133 and nestin. The susceptibility of these cells to viral infection could provide insights into the neuropathogenesis of CMV during brain development [6]. Previous studies have shown that MCMV can infect a wide variety of brain cell types including neurons and astrocytes [7]. These studies used immunohistochemical staining to demonstrate co-localization of viral antigens and cell type-specific markers. However, there is a paucity of data quantifying the effect of MCMV infection on the developing brain and which cell types involved.

Recent advances in the identification of specific neural cell types based on cell surface and intracellular markers, using flow cytometry, have lead to detailed characterization of neural stem and progenitor cells, as well as their down-stream progeny. Cell surface markers such as CD133, CD15, CD24, and CD29 have been used in a number of recently published studies [8], [9], [10]. These studies indicate that human CNS precursor cells expressing high levels of the surface antigen CD133 (CD133+/hi), with little or no CD24 (CD24−/lo), have the highest frequency of initiating clones as measured by neurosphere formation [11], [12]. Evidence suggests that these markers are also useful for characterizing similar subpopulations from the rodent CNS [13], [14]. In fact, high CD24 expression has been used to identify transit-amplifying cells [15], as well as differentiated neurons [16], and CD24 is required for terminal differentiation of neuronal progenitors [17]. Another commonly used marker is CD29, a member of the integrin family. These integrins play an important role in neural development [18], and CD29 specifically has been observed on human NSCs obtained from fetal tissue [19]. In addition, integrin signaling has been shown to be of functional relevance for both neural crest [20] and mesenchymal development [21], [22]. Finally, the antigen CD15, also known as LeX or stage-specific embryonic antigen 1 (SSEA1), has been identified as a positive selectable marker for rodent multipotent NSCs [23].

In this study we used our MCMV infection model and a multi-color flow cytometry approach to quantify the effect of MCMV on the developing brain, identifying specific target cells for viral infection and its effect on subsequent brain development. Our findings indicate that NSCs expressing CD133 and nestin are the prime target cells along with CD24(hi) neuroblasts. We also show that infection of the developing brain, which is rich in NSCs, results in reduced expression of doublecortin (DCX), a marker that identifies young/immature neurons, while the glial precursor and mature astrocyte marker, glial acidic fibrillary protein (GFAP) expression remained unaltered. Reduced DCX expression was also associated with decreased neurotrophin expression. Taken together, these results demonstrate markedly abnormal neuronal development following MCMV brain infection.

## Results

### CD133(+) cells are infected with MCMV in vivo

**Figure 1:**
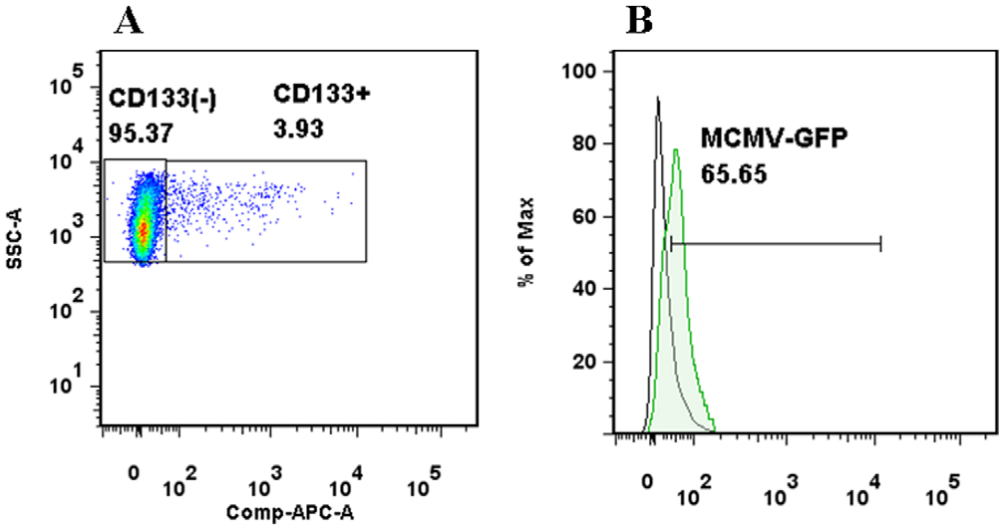
CD133(+) cells are infected with MCMV in the developing brain. **A**. Day-old neonates from timed breeders were infected intra-cerebrally (i.c.) either with 200 TCID_50_ of virulent, salivary gland passed recombinant MCMV expressing green fluorescent protein (GFP) or with mock inoculum, representing controls. A single cell suspension was prepared from the harvested brains by papain enzyme digestion at 7 d p.i. One million cells from both infected and control brains were stained for CD133, and analyzed by flow cytometry. **B**. Representative histogram showing MCMV infected CD133(+) cells, the black line represents control and the green line was obtained from MCMV-infected brain. Data shown are representative of three independent experiments using cells prepared from P7 brains obtained from 4, 3 and 5 neonates, respectively.

We have previously shown that MCMV can establish productive infection in cells which express nestin [5]. In the present study, MCMV infection of NSCs was further characterized *in vivo*. CD133(+) cells have previously been shown to be having highest frequency of initiating clones as measured by neurosphere formation [11]. We first examined if CD133(+) cells were targets for viral infection by using a recombinant MCMV expressing GFP. One-day old neonates were infected with MCMV and control littermates were mock-infected. Brain tissues from infected and mock-infected animals were harvested at 7 d p.i. for analysis by flow cytometry. Harvested brain tissue samples were digested into single cell suspension using papain as described in the methods. One million cells from infected or mock-infected mice were incubated with APC conjugated CD133 MAbs and analyzed by flow cytometry for CD133(+) cells that expressed GFP, indicating virus-infected cells. Flow cytometry analysis (Fig 1A) showed the ratio of CD133(+) cells in a P7 (post-natal day 7) brain (3.93±2.13%). We then identified GFP(+) cells that demonstrated viral infection within this CD133(+) population. We observed 65.97±3.1% of CD133+ cells were positive for GFP at 7 d p.i. (Fig. 1B). Approximately 5–6% of the total brain cells were positive for GFP (indicative of infected cells) at 7 d p.i.

### NSCs expressing nestin are targets for MCMV

**Figure 2:**
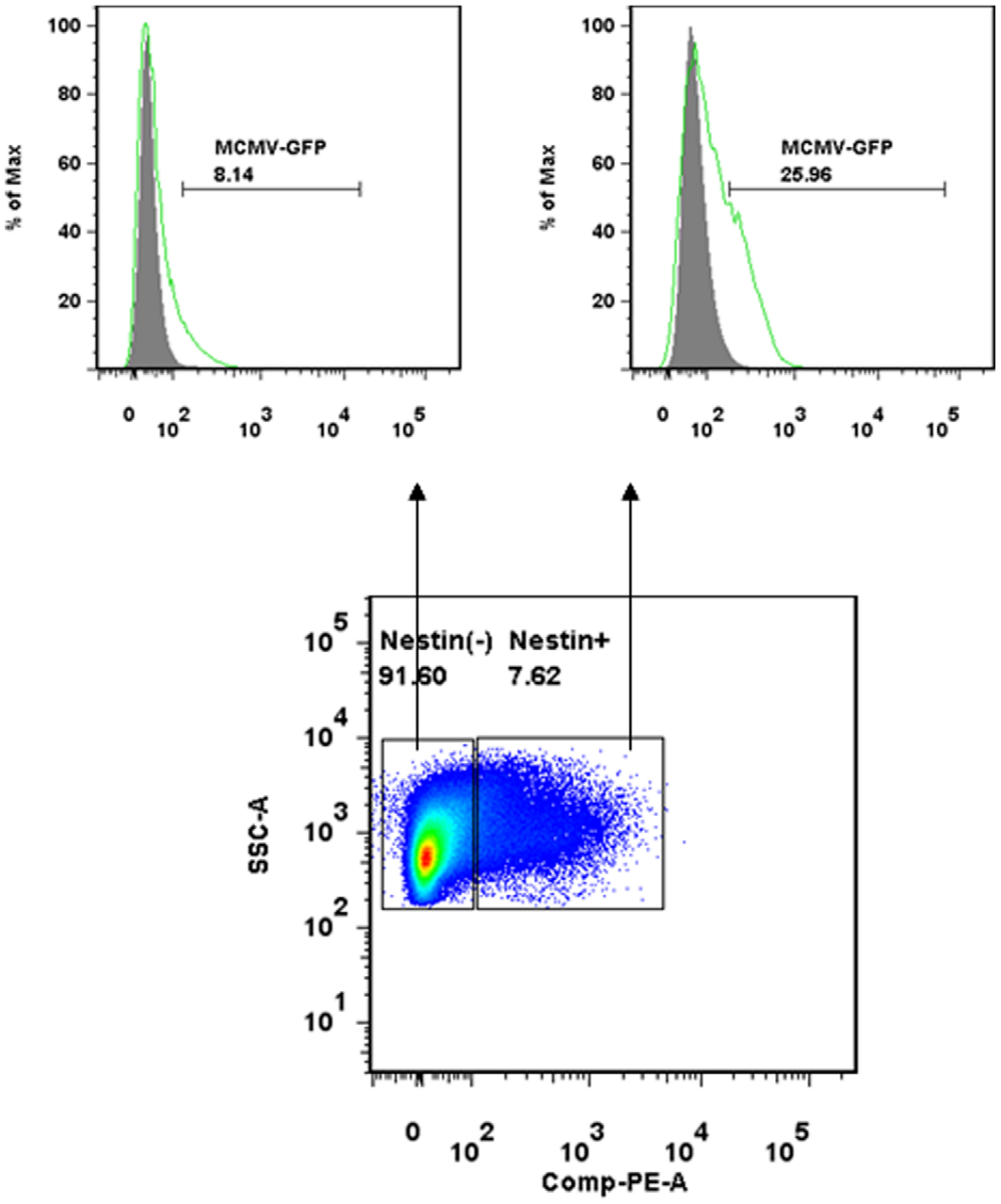
Neural stem/progentior cells (NSPC) expressing nestin are targets for MCMV. Representative dotplot showing nestin(+)/nestin(−) cells from P7 brain. Day-old neonates were infected through intra-cerebral injections with 200 TCID_50_ of recombinant virus expressing GFP and control neonates received similar dose of mock inoculum. Brain tissues from infected and control animals were harvested at 7 d p.i., and dissociated into single cells using TrypLE enzyme. The cells were stained for intracellular nestin and analyzed for nestin(−)GFP(+) as well as nestin(+)GFP(+) cells. Gates in the histograms were drawn based on mock-infected controls. Data shown are representative of three independent experiments using cells prepared from P7 brains (n = 12 neonates).

To reinforce our previous finding that MCMV preferentially infects stem cells [5], we next investigated MCMV infection in the neonatal brain, which has previously been shown to be rich in NSCs. We identified virus-infected cells using intracellular staining for the neural stem marker, nestin. Cells prepared from the brains of infected and control animals were stained for nestin and analyzed by flow cytometry. Flow cytometry analysis at 7 d p.i. demonstrated the presence of nestin(+) and nestin(−) cells from mock-infected mice (Fig 2, lower panel). There was no significant difference in the ratio of nestin (−) cells from the mock- and MCMV-infected brains. The nestin(−) and nestin(+) populations were then examined for the presence of GFP, indicative of viral infection (Fig 2, upper panel). These data clearly show that a significantly higher proportion of nestin(+) cells were found to be infected with MCMV, when compared to nestin(−) cells (26.12±2.3% versus 8.14±1.5%).

### MCMV brain infection reduces CD133(+) cell number and alters nestin expression

**Figure 3:**
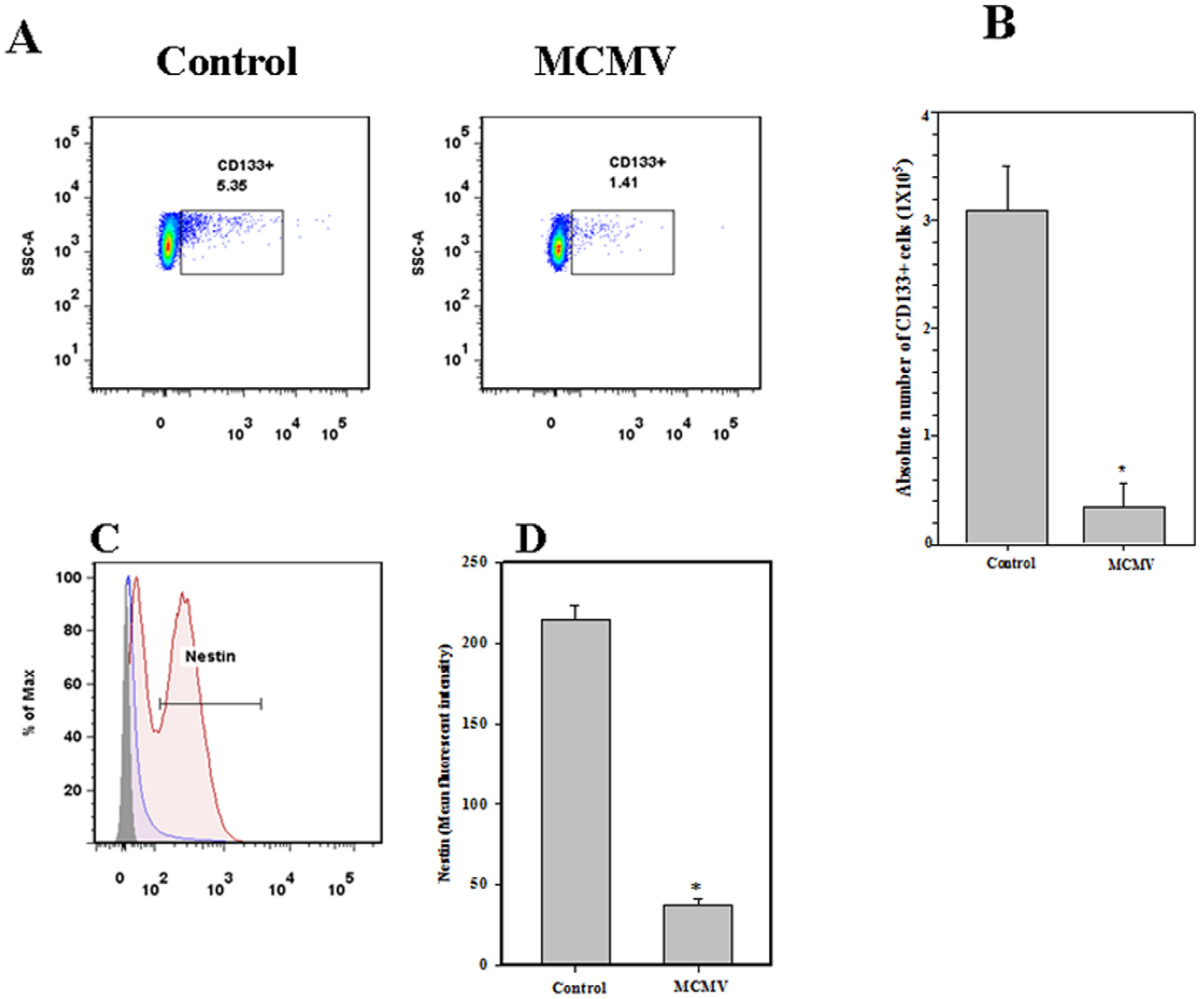
Viral infection reduces the number of CD133(+) cells and alters nestin expression in the developing brain. **A**. Representative dotplots from mock-infected and MCMV-infected brains showing CD133(+) cells at 7 d p.i. Dissociated brain-derived cells were incubated with CD133(+) Mabs and analyzed by flow cytometry. Live cell gating was performed by using 7 AAD, the live cells were also excluded from immune cells that may infiltrate during the infection using CD45-PE-Cy5. **B**. The absolute numbers of CD133(+) cells obtained from infected brains versus control brains are shown. Data were derived from 3 independent experiments, n = 3–5 neonates. *p<0.05 versus mock infected. The cells prepared from P7 brain were also stained for intracellular nestin expression. **C**. Representative histogram overlays from isotype (grey line, filled) uninfected (red line, tinge) and infected (blue line) brains. **D**. Mean fluorescent intensity for nestin expression was calculated and found to be significantly lower in the cells isolated from virus-infected brain. Data were derived from 3 independent experiments, n = 3–5 neonates. *p<0.05 versus control new born mice.

Because NSCs were found to be infected with MCMV, we then assessed whether viral infection had any effect on their number. Brain tissues were harvested from MCMV-infected and non-infected control neonates at 7 d p.i. and were stained for CD133. Flow cytometric analysis showed that numbers of CD133(+) cells were significantly reduced in the infected brains (1.41±0.80%) when compared to controls (5.35±2.0%) (Fig 3A). Absolute numbers of CD133(+) cells in control and MCMV-infected mice were 3.09×10^5^±4.1×10^4^ and 3.45×10^4^±2.1×10^4^, *p* = 0.01 (Fig 3 B). Relatively low levels of nestin expression were detected in infected-brains by flow cytometry (Fig 3C). Mean fluorescence intensity of nestin expression was found to be significantly lower among cells purified from virus-infected neonates (57.00±2.2% versus 143.00±2.84%, respectively, *p*<0.01 Student's *t* test) (Fig. 3D).

### CD24(hi)-expressing neuronal precursor cells were targets for MCMV

**Figure 4:**
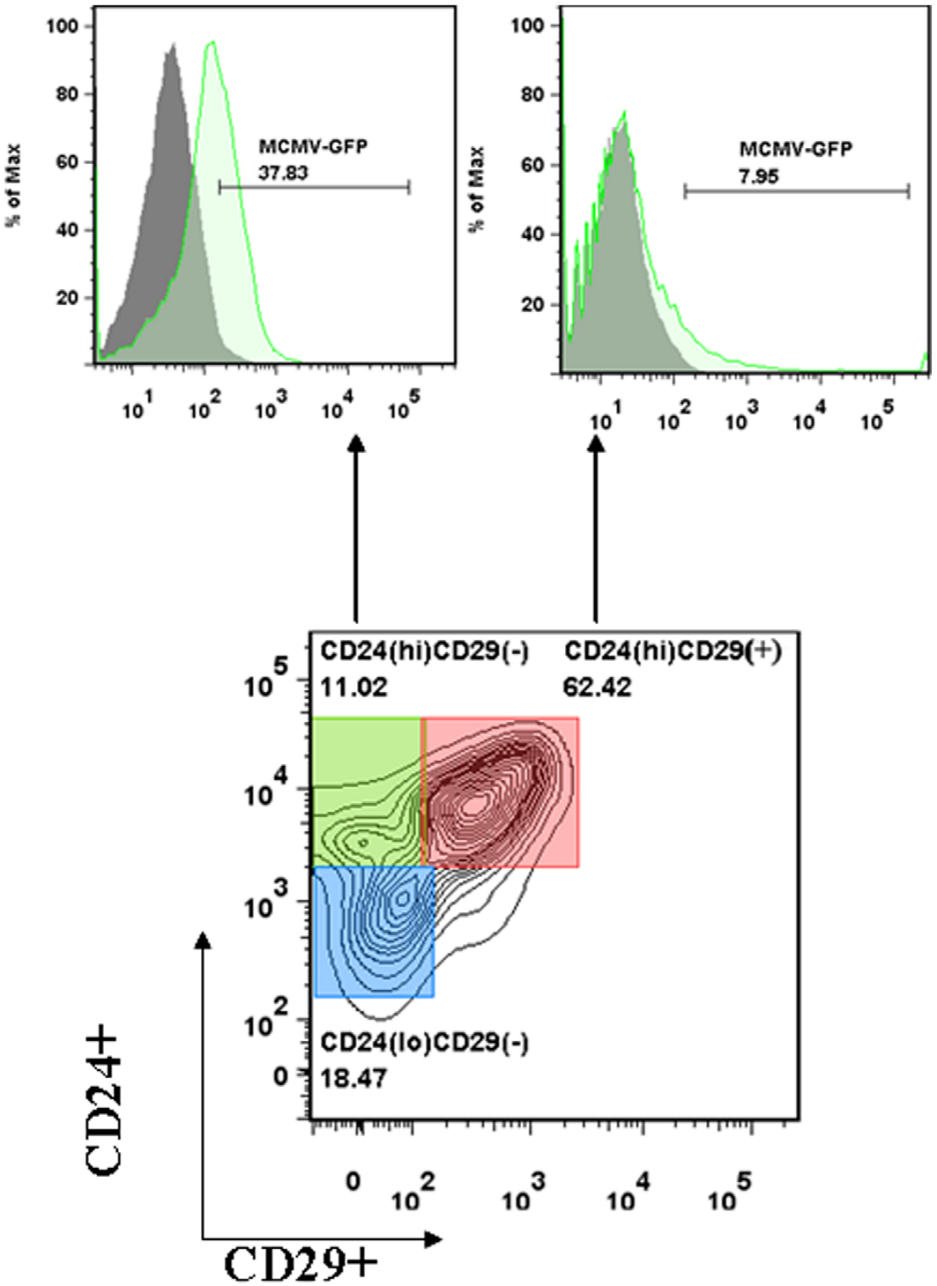
CD24(hi), neuronal precursors cells are infected with the virus. We first established a surface biomarker code based on published literature to identify the types of cells that were infected with MCMV. Brain-derived cells prepared from either infected or mock-infected animals were incubated with different Mabs for surface markers including CD15, CD24 and CD29. Representative contour plot prepared from control mice, gated on the CD45(−)CD15(−) population showing the classification of cells (lower panel) and the ratio of MCMV-GFP(+) among the subtypes are shown (upper panel). CD24 expression in the developing brain has been attributed to transit amplifying cells and is also required for terminal differentiation of neuronal progenitors. High CD29 expression is associated with neural crest like progenitor cells. Three different subtypes of cells were identified using CD24 and CD29 surface markers as shown in the representative contour plot. CD24(hi)CD29(−), CD24(hi)CD29(+) cell types were found to be infected.

To further characterize cell types infected in the developing brain, we identified cellular subsets based on their surface cluster of differentiation (CD). Previously described studies that have used CD15, CD24, and CD29 to identify various neural progenitors derived from embryonic stem cells formed the basis for our study [24]. High CD24 expression has been used to identify transit-amplifying cells [15], as well as differentiated neurons [16], and CD24 is required for terminal differentiation of neuronal progenitors [17]. The antigen CD15, also known as LeX or stage-specific embryonic antigen 1 (SSEA1), has also been identified as a positive selectable marker for rodent multipotent NSCs [23]. We went on to prepare cells from brains of virus-infected and control animals, incubated them with CD15, CD24, CD29 and CD45 MAbs, and analyzed by flow cytometry. The bottom panel of Fig. 4 shows a representative contour plot prepared from control mice, depicting three distinct cellular subsets, CD24(hi)CD29(−), CD24(hi)CD29(+) and CD24(lo)CD29(−). Gates were drawn based on the individual flurochromes and isotype control analyses. All of these subsets were negative for CD15 and CD45, indicating these subpopulations were devoid of NSCs and infiltrating immune cells, respectively. The top panels display histogram overlays from control and infected mice, showing respective GFP positive cells from each group. CD24(hi)CD29(−) cells had approximately 38.13±3.4% positive for GFP signal at 7 d p.i., while the CD24(hi)CD29(+) cells showed 8.1±2.2% of cells positive for virus. The CD24 (lo) CD29 (−) population did not show any detectable virus-infected cells, hence they were excluded from further analysis.

### Reduced numbers of proliferative CD24(hi) neuronal precursor cells in infected brains

**Figure 5:**
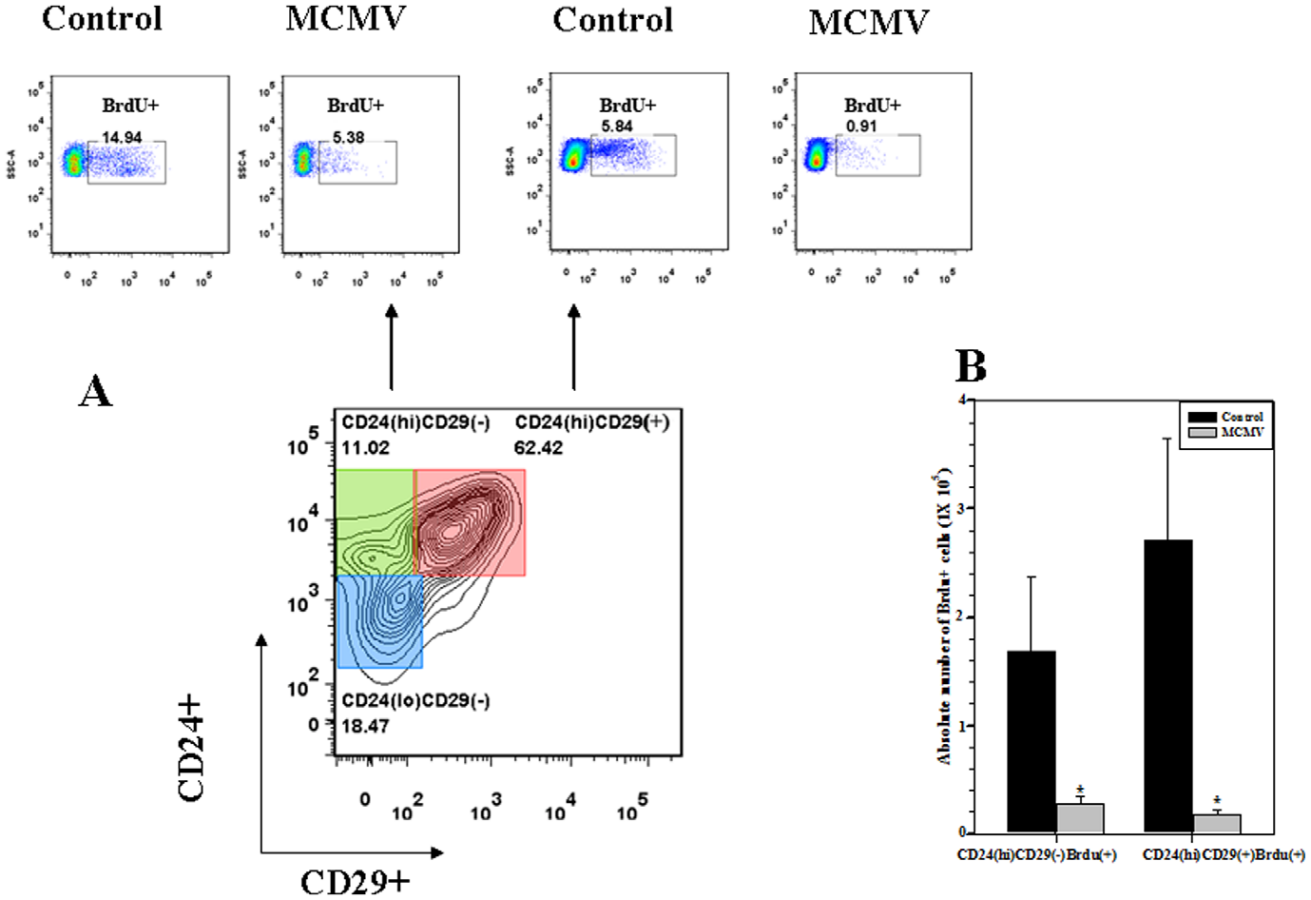
Reduced BrdU uptake by high CD24 expressing neuronal precursor cells in virus-infected brains. Mice were treated with BrdU on 7 d p.i. and 24 h later the brain samples were collected and processed for intranuclear BrdU staining as described in the methods. Cells were then analyzed by flow cytometry. **A**. Representative contour plot, gated on the CD45(−)CD15(−) population showing the classification of cells (lower panel) and the ratio of BrdU+ cells among the respective cell types is shown (upper panel). **B**. The absolute numbers of BrdU+ cells within each subtype, obtained from infected brains versus control brains are shown. Data were derived from 3 independent experiments, n = 3–5 neonates. *p<0.05 versus mock infected.

CD24 is expressed by precursors of neuronal cells in the developing brain [16] and by neuroblasts from two adult neurogenic zones: the subventricular zone (SVZ) bordering the lateral ventricle, and the dentate gyrus of the hippocampal formation [25]. We investigated the effect of virus infection on proliferation of CD24(hi) cells. Single cells prepared from brains harvested from bromodeoxyuridine (BrdU)-treated mice were first stained for surface markers such as CD24, CD29, and CD45. Intranuclear BrdU staining was done using a BrdU flow kit as described in the methods, and cells were analyzed by flow cytometry. The individual subsets of cells that were previously defined were analyzed for BrdU+ (Fig. 5A). In these studies, we observed a significant decrease in the number of CD24(hi)CD29(−)BrdU(+) and CD24(hi)CD29(+)BrdU(+) compared to control mice (2.81×10^4^±6.2×10^3^ and 1.73×10^4^±5.3×10^3^±5.3×10^3^ versus 1.68×10^5^±6.9×10^4^ and 2.7×10^5^±9.3×10^4^ in control animals, respectively, *p*<0.01 Student's *t* test) (Fig. 5B).

### Altered expression of the Oct4 transcription factor

**Figure 6:**
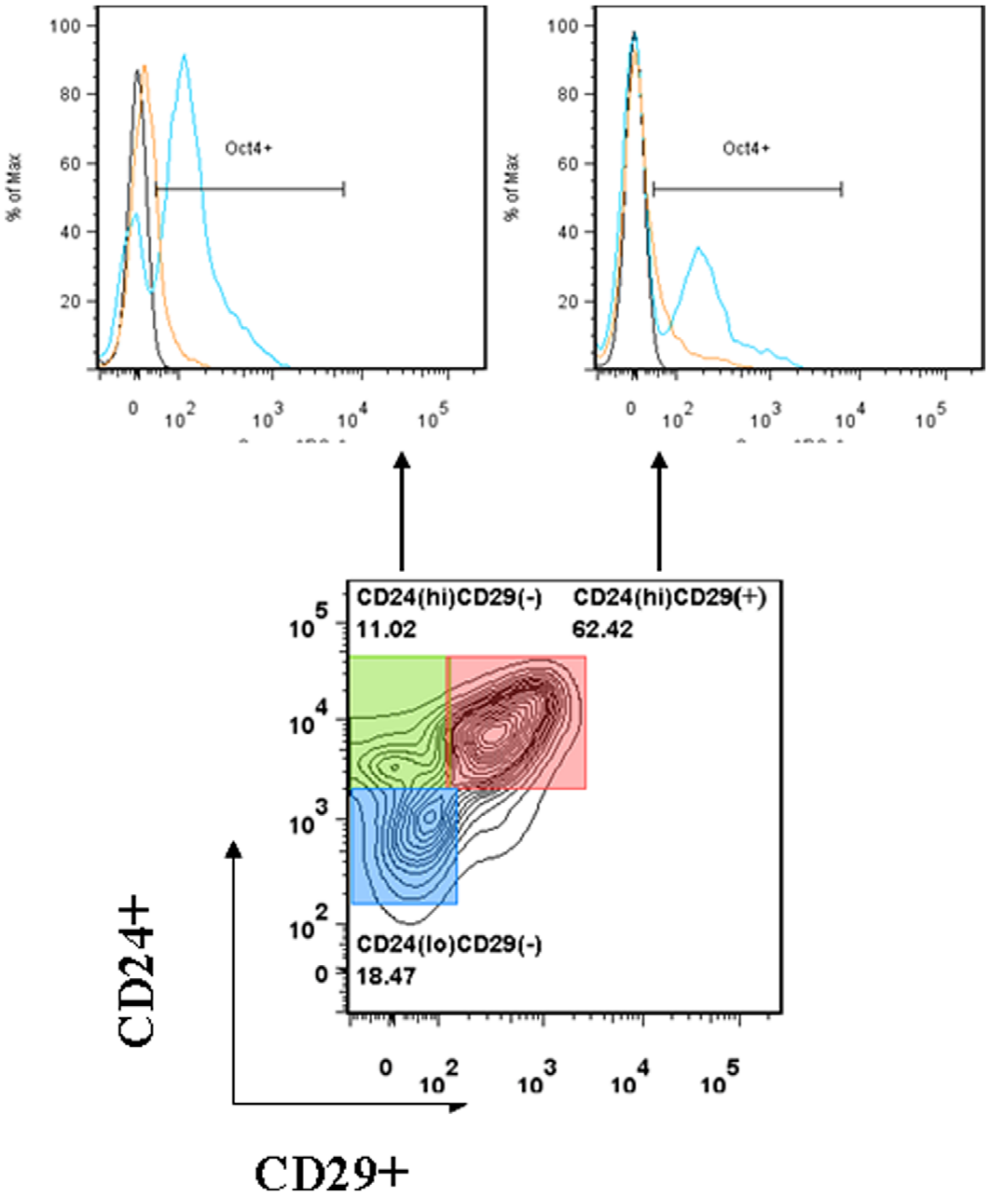
Differential expression of the transcription factor Oct4 in infected brains. Brain-derived cells prepared from either infected or mock-infected animals were incubated with different MAbs for surface markers including CD15, CD24, and CD29. The cellular subsets were then analyzed for expression of the transcription factor Oct4 and compared with mock-infected brains in the histogram overlays. Histogram overlays show a blue line representing control neonates, an orange line MCMV infected, and a black line representing isotype for Oct4 staining. Data were derived from 3 independent experiments, n = 3–5 neonates.

Oct4 is critically involved in self-renewal of embryonic stem cells, so it is frequently used as a marker for undifferentiated cells. Oct4 expression must be closely regulated; too much or too little will actually induce differentiation [26]. The transcription factors Oct4, Sox2, and Nanog are capable of inducing the expression of each other, and are essential for maintaining the self-renewing undifferentiated state of the inner cell mass of the blastocyst, as well as in embryonic stem cells [27]. It has been previously shown that CD24 cells express Sox2 indicating they still retain stemness [28]. To determine if CD24(hi) cells from the developing brain expressed Oct4, we performed intracellular staining for Oct4 as well as surface stained for CD15, CD24 and CD29 and analyzed these cell populations using flow cytometry. In this study, we demonstrated that CD24(hi)CD29(−) and CD24(hi)CD29(+) cells, isolated from uninfected control brains, expressed Oct4 (Fig. 6, upper panel, histogram with blue line). Although CD24(hi)CD29(−) negative cells were found to express Oct4, it appeared that once CD29 expression occurred on these CD24(hi) cells the number of Oct4-expressing cells was reduced (Fig. 6, upper panel, histogram on the right). We then went on to determine if MCMV infection had any effect on Oct4 expression among this defined subset of cells. The upper panel shows histogram overlays for Oct4 staining from brain cells isolated from control and infected brains, as well as isotype control staining. Data obtained from these experiments demonstrated that Oct4 expression was reduced in cells obtained from virus-infected brains compared to the cells from the brains of uninfected animals (upper panel, histogram overlays).

### Expression of doublecortin is decreased following MCMV brain infection

**Figure 7:**
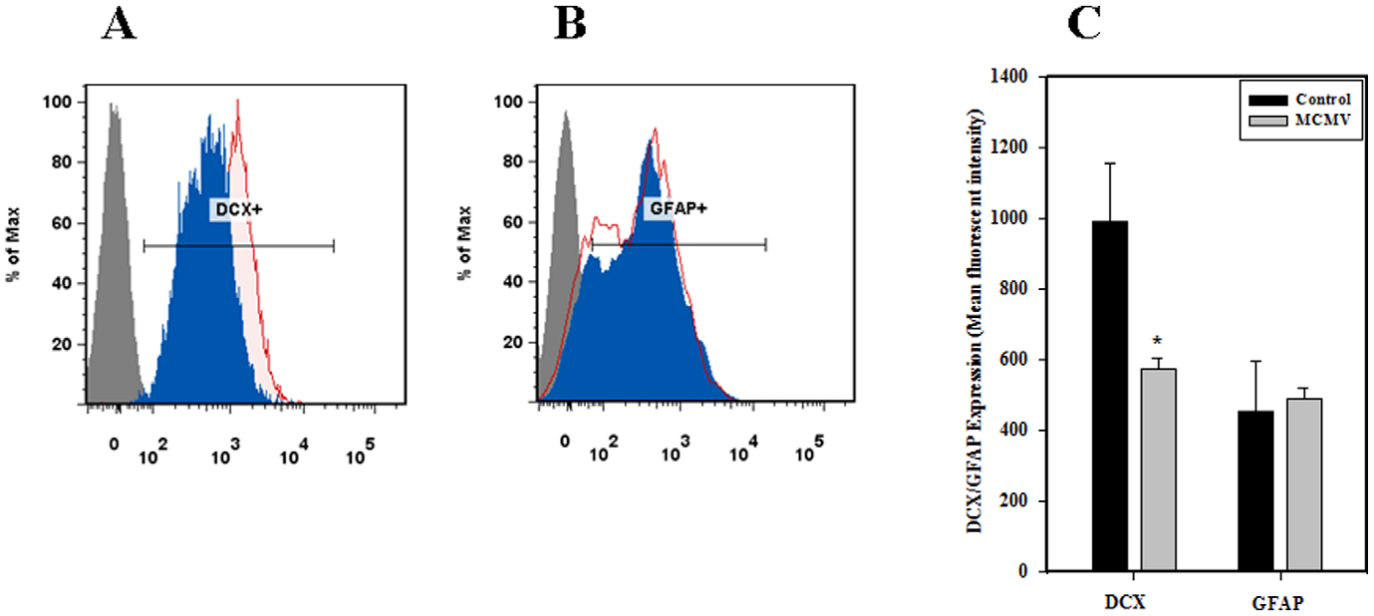
Decreased expression of DCX in the infected brain. Cells isolated from MCMV-infected and control brains were analyzed for intracellular doublecortin (DCX) and glial fibrillary acidic protein (GFAP) at 7 d p.i. Histogram overlays from isotype (grey line, filled), infected (blue line, filled), and control (red line, tinge) are shown for: **A**. DCX, a marker for young/immature neurons and **B**. GFAP, a marker for glial precursors. Infected brains showed reduced expression levels of intracellular DCX compared to control brains while there was no difference in the expression levels of GFAP in MCMV infected versus control brains. **C**. Data were derived from 3 independent experiments, n = 3–5 neonates. *p<0.05 versus mock infected.

We next examined if viral infection is associated with abnormal expression of structural proteins such as doublecortin (DCX) and GFAP. DCX is a microtubule associated protein expressed by neuroblasts and is accepted as an effective read-out for neurogenesis. GFAP is an intermediate filament protein that is thought to be specific for astrocytes. Using an intracellular staining technique and flow cytometry, we found that expression of DCX is altered in the virus-infected brain (Fig. 7A), while GFAP expression remained unaltered (Fig. 7B). Histogram overlays are shown for both DCX and GFAP expression and were prepared from isotype, control, and virus infected peaks. Mean fluorescence intensity of DCX and GFAP expression were also measured between the groups. Expression levels of DCX in virus-infected brains were found to be significantly lower compared to uninfected control animals (511±140% versus, 1178±161% respectively, *p*<0.05 Student's *t* test) and there was no significant difference in the MFI of GFAP expression among groups studied (Fig. 7C).

### Viral brain infection down-regulates BDNF and NT3 levels

**Figure 8:**
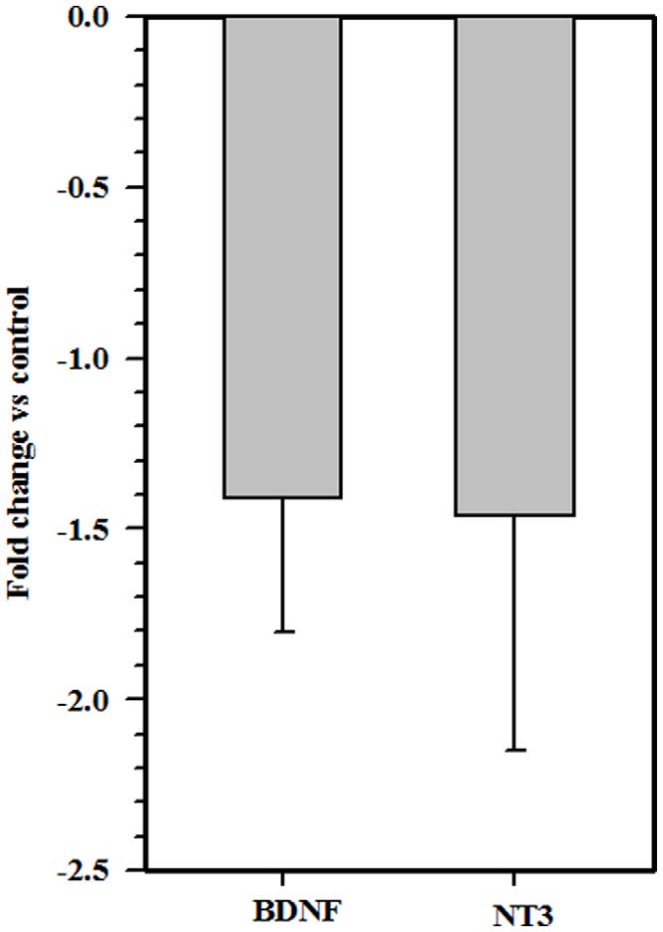
MCMV brain infection of the neonates down-regulates neurotrphin expression levels. BDNF and NT3 mRNA expression was assessed using quantitative real-time RT-PCR on total RNA extracted from whole brain homogenates of control and virus- infected neonates at 7 d p.i. mRNA levels were normalized to HPRT and are presented as mean ± SD normalized expression from pooled data obtained using 3–5 animals per group from 2 independent experiments.

Brain-derived neurotrophic factor (BDNF) and neurotrophin 3 (NT3) have been shown to play a role in the development of the CNS. These two neurotrophin molecules are also known to be important in post-natal cerebellar development [29], [30]. We went on to determine mRNA levels for these two neurotrophins in control and virus-infected brains at 7 d p.i. using quantitative real time PCR. In these experiments, mRNA levels of both BDNF and NT3 were markedly down-regulated in virus-infected neonatal brains when compared to control mice (Fig. 8).

## Discussion

In this study we found that NSCs and neuronal precursor cells are the principal target cells for MCMV within the developing brain. Additionally, viral infection caused a marked loss of NSCs expressing CD133 and nestin. We also showed that infection of neonatal brain leads to abnormal development as indicated by loss of CD24 (hi) cells that incorporated BrdU. The infection of neonatal brain was also associated with altered expression of the neurotrophins BDNF and NT3, which are essential for normal development of brain [29], [30]. “Additionally” we found decreased expression of doublecortin, a marker to identify young neurons, following viral brain infection.

NSCs are abundant in the fetal brain and the increased susceptibility of the fetus to viral infection may explain the predominance of neurological damage associated with congenital CMV infection. Previous studies have shown that intracranial inoculation of MCMV results in wide spread brain infection [31], [32]. In our previous report [5], we showed widespread MCMV brain infection in adult mice, particularly in cells of the periventricular zones as well as regions of the brain in direct contact with cerebrospinal fluid, strikingly similar to descriptions of human CMV (HCMV) ventriculoencephalitis in AIDS patients and infants with severe CNS manifestations of congenital HCMV infection. In this study, intracranial inoculation resulted in wide-spread infection within the brain. GFP signal was detected in various regions including cerebral cortex, olfactory bulb, cerebellum and brain stem, “however” the infection was most profound around the ventricles (Fig. S1). In contrast to immunocompetent adult mice, neonates infected intracranially failed to control the infection and succumbed to infection starting day 12 p.i. with 200 TCID50 (Fig. S2).

NSCs exhibit extensive self-renewal and multipotency (i.e., the ability to generate neurons and glial cells). Neurogenesis continues beyond embryonic life and postnatal and adult neurogenesis have been postulated to have critical roles in learning, memory, and cognitive development [33], [34]. In mice, much of the brain development takes place postnatally. Interestingly, we observed that there was no significant difference in expression levels of CD24 (hi) and nestin at embryonation day (ED) 14.5 and post-natal day 7 (Fig. S3). These newborn mice also failed to mount effective adaptive immune response and the immune infiltrate predominantly consisted of macrophages with activated resident microglia (Fig. S4).

Neurotropic viruses (e.g., HIV) disturb the normal adult neurogenesis pattern, a possible cause for development of dementia in these patients. More importantly, it has been demonstrated that HIV infects NPCs and leads to quiescence in these cells [35], [36]. Other neurotropic viruses like Japanese encephalitis virus (JE virus), which also targets the CNS, infects embryonic NPCs, replicates in these cells, inhibits their growth, and decreases proliferation [37]. Closely resembling these findings, we report here, that MCMV infects NSCs in the developing brain and reduces their number *in vivo*. Using flow cytometry, along with a recombinant GFP-expressing MCMV, it was possible to quantify this viral brain infection and determine the number of highly susceptible NSCs *in vivo*. Our findings also indicated that CD133+ cells were the major target cells for MCMV, compared to other cell types that were studied.

There is considerable analytical value in the identification and isolation of multiple neural subsets by their expression of surface antigens using flow cytometry. Fluorescence-activated cell sorting (FACS) has been successfully utilized in sorting cells based on these surface markers and it has high scientific value for the fields of regenerative medicine, and stem cell biology [9], [38], [39]. The combinatorial detection of surface markers by multicolor flow cytometry has been widely applied in the fields of hematology and immunology [40], [41], but has up to now only been marginally exploited in neurobiology [12], [42]. Surface markers such as CD15, CD24, and CD29 have been described to study neural lineage cells derived from pluripotent stem cells [24]. Based on this finding, we utilized the identical markers to identify cells that were obtained from digestion of mouse brain tissue. Using these methods, we were able to identify 3 distinct cellular populations; CD24(hi)CD29(−), CD24(hi)CD29(+) and CD24(lo)CD29(−). Within these subsets we identified virus-infected cells and observed that the majority of CD24(hi) cells were infected. High CD24 expression has been used to identify transit-amplifying cells [15], as well as differentiated neurons [16], and CD24 is required for terminal differentiation of neuronal progenitors [17]. Infection of CD24(hi) cells demonstrates that neuronal precursor cells are highly susceptible to infection with MCMV. Interestingly, we were unable to detect viral infection in cells that expressed CD24 at low levels. It is well documented in the literature that hi CD24 expression is associated with neuronal precursor cells or neuroblasts. Based on the available literature, it is believed that CD24(hi)CD29(−), CD24(hi)CD29(+), and CD24(lo)CD29(−) cells would give rise to neurons, a mixture of neurons and glia, and identify mature neurons, respectively [24]. Our findings indicated that neuronal precursors expressing hi CD24 are predominant target cells for the virus. In certain cancer cell lines, it has been shown that CD133+ cells were identified as cancer stem cells which would also react with CD29 [43], however in our study we did not find any such correlation between CD133 and CD29 among the cells that we studied.

Sustained proliferation is a key feature of neural precursor cells and it has been shown that viruses like JE virus and HIV can induce quiescence in these cells by impairing their proliferative ability [35], [37]. Consistent with these findings, we observed that CD24(hi)CD29(−) and CD24(hi)CD29(+) cells from control mice were found to incorporate BrdU, with the latter subset being more efficient. The ability of CD24(hi) cells to incorporate BrdU was remarkably decreased in virus-infected brains, suggesting that MCMV inhibits DNA synthesis in these CD24(hi) proliferative cells. In this study, we also report down-regulation of the multipotency marker, Oct4 within the above defined subset of cells. This may have deleterious effects on normal brain development.

Having observed that CD24(hi) cells were extensively infected and had decreased ability to proliferate, we then sought to identify the effect of viral infection on cells that are down-stream of CD24-expressing neuronal precursors. Our analysis for intracellular DCX, a marker for young neurons, was found to be significantly down-regulated in virus-infected brains. DCX plays an important role in signaling for neuronal migration during brain development and is a marker of early migratory neuroblasts. DCX haploinsufficiency can lead to various degrees of mental retardation, the extent of retardation is linked to the quantity of arrested neurons in the white matter [44], [45]. This has been previously reported in *in vitro* studies using human neural stem/precursor cells that were infected with CMV, the DCX expression was down-regulated, shown at both mRNA and protein levels [46]. On the other hand, we did not observe any change in the expression levels of intracellular GFAP, a marker for astrocytes. HCMV infection of neural progenitor cells in the undifferentiated stage down-regulated GFAP expression while it remained unaltered following infection after differentiation [46]. Further validation of proliferation arrest in neuronal precursor cells and reduced DCX expression with progressive infection was obtained from decreased expression of neurotrophins, such as BDNF and NT3. The activities of BDNF are pleiotropic and include protection from neural apoptosis, enhanced neuronal proliferation, increased granular neuron migration, and long-term potentiation [47], [48]. Down-regulation of the gene encoding of the neurotrophin NT3 was also associated with viral brain infection.

Infection of different resident cells of the CNS and loss of neuroepithelial cells secondary to lytic infection have also been reported [49], [50]. Although MCMV targets several other cell types in the developing brain, NSCs in particular bear the brunt of virus infection. In conclusion, we found that MCMV brain infection of new born mice causes significant loss of NSCs, decreased proliferation of neuronal precursor cells and loss of young neurons expressing DCX. This neuronal loss was associated with down-regulation of multipotency marker, Oct4 and neurotrophins, indicating abnormal brain development.

## Materials and Methods

### Ethical statement

The animal use protocols used were approved by the University of Minnesota Institutional Animal Care and Use Committee (Protocol Number: 0807A40181).

### Virus and animals

A recombinant MCMV that expresses green fluorescent protein (GFP) under control of the human elongation factor-1a promoter, inserted at the immediate-early gene (IE2) site (strain K181 MC.55 [ie2^−^ GFP^+^]) was kindly provided by Jon Reuter [50], [51]. 200 TCID_50_ of virulent, salivary gland–passaged, sucrose gradient–purified virus was used for all intracerebral (i.c.) infections. The GFP-expressing virus was expanded on NIH 3T3 mouse fibroblasts and purified by centrifugation over a sucrose cushion. Mice were purchased from The Jackson Laboratory or Charles River Corporation. Mating pairs were setup and carefully monitored each day until they gave birth. All animals were maintained in a specific pathogen–free facility.

### Intracerebral infection of neonatal mice

Intracerebral infection of neonatal mice was performed as previously described [52]. Briefly, neonatal mice were placed on ice for 3 min to induce anesthesia before being secured in a cooled, stereotaxic frame (Stoelting) maintained at 4°C to 8°C by a dry ice/ethanol reservoir. A 10 µL syringe (Hamilton Company) fitted with a 30 gauge hypodermic needle (Hamilton Company) was used to inject virus (200 TCID_50_ in 2 µl) or saline into the right lateral cerebral lobe. No incision was made for injection. The neonatal skull was penetrated with the needle for all injections.

### Preparation of single cell preparation from neonatal brains and flow cytometry

Entire brain tissues obtained from control and infected neonates at 7 d p.i. were dissociated into single cell suspension as using previously described methods [10]. Briefly, tissue samples were gently minced using a scalpel and were resuspended in a purified trypsin-like replacement in Dulbecco's PBS/EDTA (TrypLE Select; Invitrogen; containing 200 units/mL DNase I (Roche) and 1 mM MgCl2. We also used the enzyme papain (12 units/mL; Worthington, Lakewood, NJ) in experiments that involved staining for CD133. The enzyme papain was preactivated in 1.1 mM EDTA, 0.067 mM mercaptoethanol, and 5.5 mM cysteine-HCl for 30 min before addition. The absence of bicarbonate in the HBSS allowed dissections and incubations to be performed in a room atmosphere. Samples were placed in a 37°C water bath for 30 min during digestion. Samples were then spun at 200×g for 5 min, resuspended in fresh HBSS/DNase/MgCl2 without enzyme, and filtered using cell strainers (40 µm). Cells were counted by trypan blue dye exclusion method, 1×10^6^ cells from both control and infected brains were used for flow cytometric analysis.

For flow cytometry, cells were resuspended in flow cytometry buffer, consisting of 1× PBS, pH 7.2, containing 2% fetal bovine serum. Cells were counted and diluted to a density of 10^6^ cells per milliliter. For surface marker analysis, cells were stained with anti-mouse cell surface markers for 30 min at 4°C. Antibodies used were, CD133-APC or PE (Miltenyi Biotec Inc, Auburn, CA), CD15-APC or PE (ebiosciences, San Diego, CA), CD24-PE-Cy7, CD45-PE-Cy5, CD29-PE (BD Biosciences, San Jose, CA). For staining intracellular markers, cells were surface stained, prior to fixation and permeabalization using Cytofix/Cytoperm (BD Biosciences, San Jose, CA). Surface markers were selected based on the experiment being performed: nestin-PE or APC (R&D Systems, Minneapolis, MN and BD Biosciences, San Jose, CA), DCX, GFAP-PE (Santa Cruz Biotechnology, Santa Cruz, CA) and Oct4-APC (R&D Systems, Minneapolis, MN). Cells were washed in staining buffer, and then secondary fluorescent-conjugated antibody (if needed) was added at the appropriate dilution and incubated on ice for 30 min. Cells were analyzed on a FACSCanto flow cytometer (BD Biosciences). Background fluorescence was measured using unlabeled cells and cells labeled with isotype control or secondary antibody alone; and used to set gating parameters between positive and negative cell populations. Cell aggregates and small debris were excluded from analysis or isolation on the basis of side scatter and forward scatter; dead cells (7 AAD+) and CD45-PE-Cy5+ immune cells were excluded from analysis. The data collected were analyzed using FlowJo software (TreeStar).

### BrdU labeling and detection of BrdU+ cells by flow cytometry

BrdU labeling of neonatal mice was done as described previously [53], MCMV-infected or control newborn mice were injected intraperitoneally with BrdU (50 µg/g body weight) 7 d after infection. Mice were killed at 24 h after BrdU administration. Single cell suspension was prepared from control and infected brains as described earlier. Cells were surface stained with CD24-PE-Cy7 and CD29-PE, and with CD133-APC. Intranuclear BrdU was stained using the FITC BrdU Flow Kit (BD Biosciences San Jose, CA). Interference of MCMV-GFP signal with FITC BrdU was ruled out as repeated fixing and permeablization during the staining protocol dampened the GFP signal from virus-infected cells, this was confirmed on simultaneously treated cells by fluorescent microscopy for GFP signal prior to staining with BrdU-FITC. The cells were fixed and permeablized by resuspending in 100 µL of Cytofix/Cytoperm (BD Biosciences) buffer at room temperature for 30 min, followed by the addition of 1 mL of wash buffer (BD Biosciences). The samples were spun at 300×*g* for 5 min and the supernatant was aspirated. The cells were then resuspended in 100 µl of Cytoperm Plus buffer (BD Biosciences) on ice for 10 min. After washing and centrifuging, the cells were resuspended in 100 µl of Cytofix/Cytoperm buffer at room temperature for 5 min. The cells were then resuspended in 100 µL of DNAse (30 µg; stock from kit was diluted in PBS (Ca^2+^/Mg^2+^ free) containing 0.1 mM CaCl_2_ and 10 mM MgCl_2_) in a dry heat block at 37°C for 1 h. Following washing and centrifuging, the cells were resuspended in 50 µl of FITC-conjugated anti-BrdU (1∶50 dilution) in the dark, at room temperature for 20 min. After the samples were washed, they were resuspended with 20 µl of the nuclear marker, 7-AAD, at room temperature in the dark. The cells were then resuspended in 1 mL of staining buffer (PBS, 3% FBS, 0.09% sodium azide). Prior to analysis, cells were filtered through a cell strainer cap (30 µm) to remove debris. The data was collected the same day on a BD FACSCanto system. The data collected was analyzed using FlowJo software (TreeStar).

### Real-time PCR for BDNF and NT3

Total RNA was extracted from brain tissue homogenates with Trizol reagent (Invitrogen, Carlsbad, CA). One µg RNA was DNase (Ambion, Applied Biosystems, Austin, TX) treated and reverse transcribed to cDNA with SuperScript™ III (Invitrogen), dNTP (GE Healthcare, Piscataway, NJ) and oligo (dT)_12–18_ (Promega, Madison, WI). Real-time PCR was performed in Mx3000p (Stratagene, La Jolla, CA) with SYBR Advantage qPCR Premix (Clontech, Mountain View, CA), primers and cDNA according to manufacturer's protocol. Reaction conditions for qPCR were as follows: initial denaturation at 95°C for 15 sec, amplification for 40 cycles at 95°C for 10 sec, 60°C for 10 sec and 72°C for 10 sec followed by dissociation curve analysis (1 cycle at 95°C for 60 sec, 55°C for 30 sec and 95°C for 30 sec) to verify PCR product specificity. Primer sequences used were: sense 5′-GGTATCCAAAGGCCAACTGA-3′ and antisense 5′-CTTATGAATCGCCAGCCAAT-3′ for BDNF; sense 5′- CCAGGCGGATATCTTGAAAA-3′ and antisense 5′-AGCGTCTCTGTTGCCGTAGT –3′ for NT3; sense 5′-TGCTCGAGATGTCATGAAGG-3′ and antisense 5′-AATCCAGCAGGTCAGCAAAG-3′, for hypoxanthine guanine phosphoribosyl transferase (HPRT)-1 as housekeeping gene. After normalizing to HPRT-1 expression (ΔCt = target gene Ct−HPRT Ct) and then to control group (ΔΔCt = treatment ΔCt−C ΔCt), relative quantification using 2∧^−ΔΔCt^ was calculated as fold change of target mRNA expression vs. control.

## Supporting Information

Figure S1 Periventricular cells are preferentially infected. Coronal sections from neonatal brains showing GFP-expressing cells indicative of viral infection with recombinant MCMV at 7 d p.i. **A**. Lower magnifications demonstrate that MCMV is localized to cells surrounding the ventricles. **B**. Adjacent serial sections, showing GFP+ cells stained for nucleus (DAPI).
(TIFF)

Figure S2 Neonatal mice fail to control viral brain infection. Day-old littermates were injected intracranially with MCMV (500 or 200 TCID_50_ in 2 µl) or with saline. The infected and control neonates were nourished in identical conditions. Data are expressed as percent survival in each group at the indicated time point, followed over the 15 d time-course of the experiment.
(TIFF)

Figure S3 Experimental model simulates congenital cytomegalovirus infection. Embryos from timed breeders were collected at ED14.5 and brains were dissected out to prepare single cell suspension. Cells were also prepared from brains harvested from 7 d old neonates. Cells were surface stained for CD24 and also for intracellular nestin. Representative dotplot and histogram showing ratios of CD24(hi) cells and percent of maximum cells expressing nestin from both **A**. ED14.5 and **B**. P7 brains are shown. No significant difference in the expression levels of CD24 and nestin was observed between the groups analyzed. Data are derived from two independent experiments, n = 3–5 embryos/neonates.
(TIFF)

Figure S4 Immune responses to MCMV brain infection predominantly consist of macrophages. At 7 d p.i., leukocytes were isolated from MCMV-infected neonatal brains. Brain tissues harvested from 4–6 animals were minced finely in RPMI (2 g/L D-glucose and 10mM HEPES) and mechanically disrupted (in Ca/Mg free HBSS) at room temperature for 20 min. Single cell preparations from infected brains were resuspended in 30% Percoll and banded on a 70% Percoll cushion at 900×g at 15°C. Brain leukocytes obtained from the 30–70% Percoll interface were stained with anti-mouse immune cell surface markers for 45 min at 4°C (CD45-PE-Cy7, CD11b-APC-CY7, Ly-6G-FITC, MHC Class II- PE, F4/80-APC, CD4-FITC and CD8-PE BD Biosciences, San Jose, CA, multiple set of analyses were performed to accommodate different combination of markers with fluorochromes) and analyzed by flow cytometry using a BD FACSCanto. Live leukocytes were gated using forward scatter and side scatter parameters and analyzed using FlowJo software (TreeStar, Inc.). **A**. We identified four distinct populations as shown. **B**. Histogram showing F4/80+ cells from CD45(hi)CD11b(hi) cells. **C**. Histogram showing MHC class II+ cells, indicating microglial activation from CD45(int)CD11b(+). **D**. Dotplot showing ratio of CD4 and CD8 from CD45(hi)CD11b(−) and from CD45(hi)CD11b(+).
(TIFF)
