# Supplementary figures and images for: Murine Cytomegalovirus Infection of Neural Stem Cells Alters Neurogenesis in the Developing Brain
Source: PLoS One. 2011 Jan 13;6(1):e16211. doi: 10.1371/journal.pone.0016211 (PMC3020957; doi:10.1371/journal.pone.0016211)

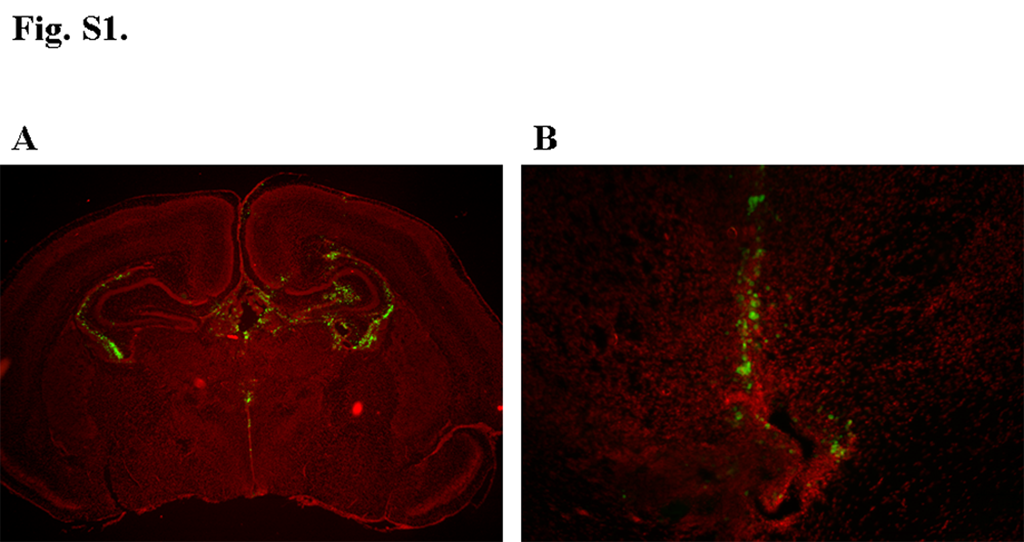

Supplement: Figure S1 — Periventricular cells are preferentially infected. Coronal sections from neonatal brains showing GFP-expressing cells indicative of viral infection with recombinant MCMV at 7 d p.i. A. Lower magnifications demonstrate that MCMV is localized to cells surrounding the ventricles. B. Adjacent serial sections, showing GFP+ cells stained for nucleus (DAPI). (TIFF) [file pone.0016211.s001.tiff]

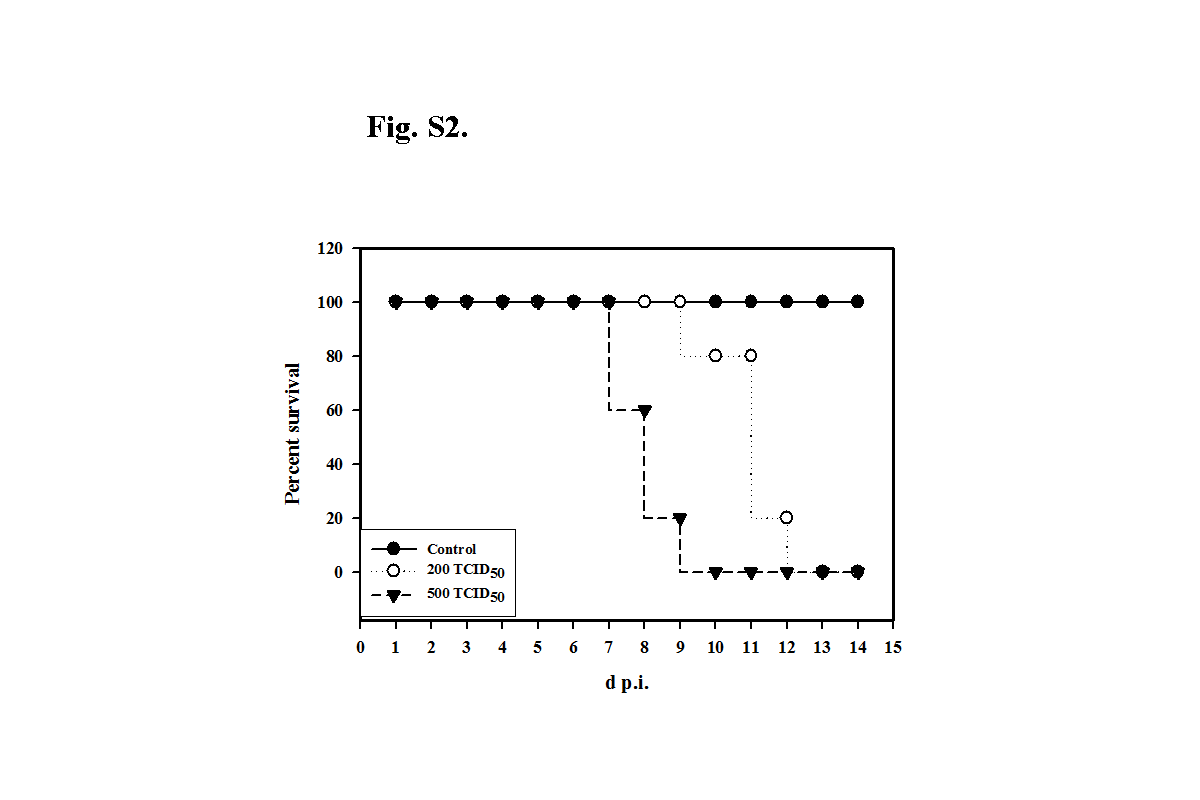

Supplement: Figure S2 — Neonatal mice fail to control viral brain infection. Day-old littermates were injected intracranially with MCMV (500 or 200 TCID50 in 2 µl) or with saline. The infected and control neonates were nourished in identical conditions. Data are expressed as percent survival in each group at the indicated time point, followed over the 15 d time-course of the experiment. (TIFF) [file pone.0016211.s002.tiff]

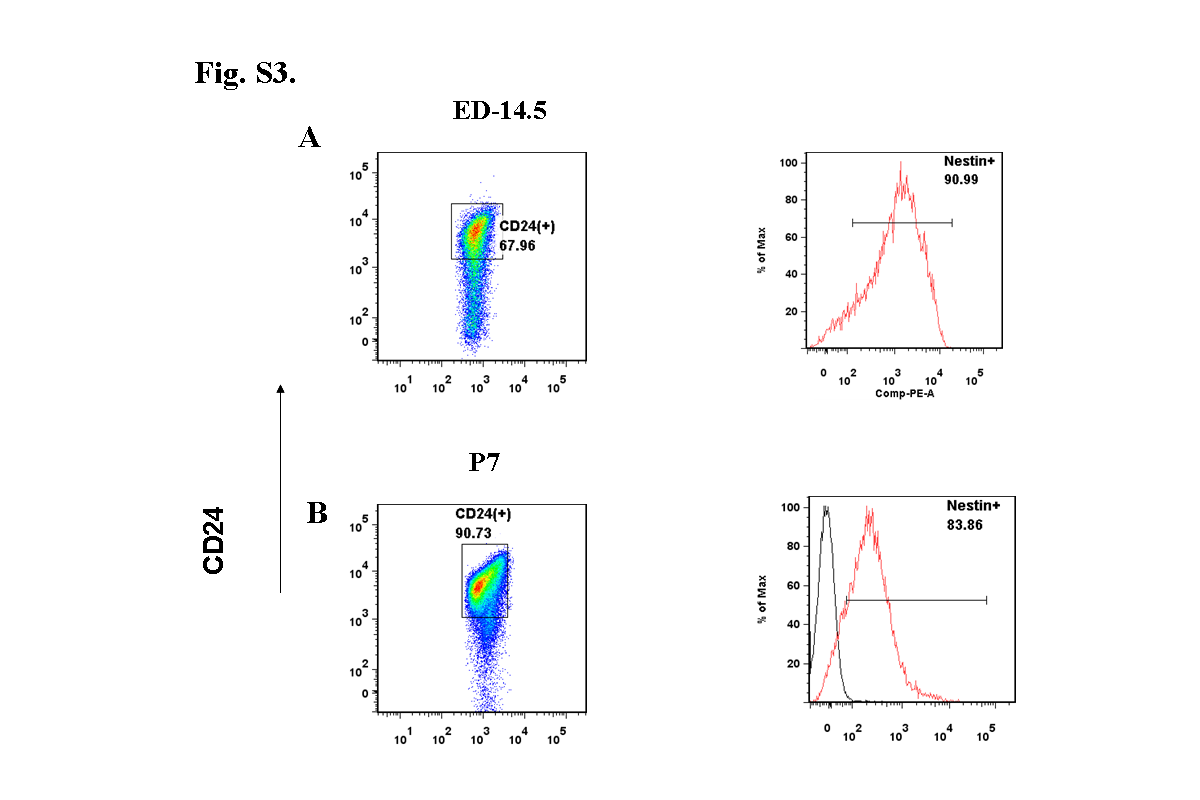

Supplement: Figure S3 — Experimental model simulates congenital cytomegalovirus infection. Embryos from timed breeders were collected at ED14.5 and brains were dissected out to prepare single cell suspension. Cells were also prepared from brains harvested from 7 d old neonates. Cells were surface stained for CD24 and also for intracellular nestin. Representative dotplot and histogram showing ratios of CD24(hi) cells and percent of maximum cells expressing nestin from both A. ED14.5 and B. P7 brains are shown. No significant difference in the expression levels of CD24 and nestin was observed between the groups analyzed. Data are derived from two independent experiments, n = 3–5 embryos/neonates. (TIFF) [file pone.0016211.s003.tiff]

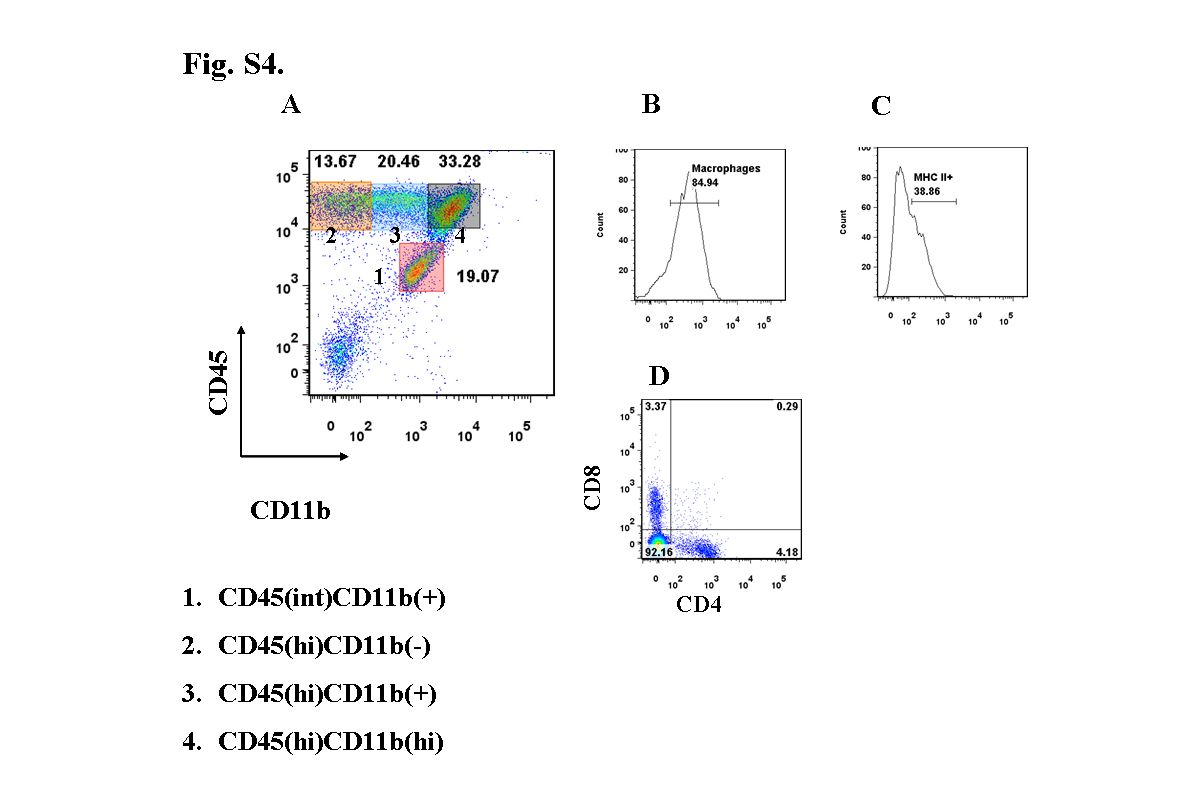

Supplement: Figure S4 — Immune responses to MCMV brain infection predominantly consist of macrophages. At 7 d p.i., leukocytes were isolated from MCMV-infected neonatal brains. Brain tissues harvested from 4–6 animals were minced finely in RPMI (2 g/L D-glucose and 10mM HEPES) and mechanically disrupted (in Ca/Mg free HBSS) at room temperature for 20 min. Single cell preparations from infected brains were resuspended in 30% Percoll and banded on a 70% Percoll cushion at 900×g at 15°C. Brain leukocytes obtained from the 30–70% Percoll interface were stained with anti-mouse immune cell surface markers for 45 min at 4°C (CD45-PE-Cy7, CD11b-APC-CY7, Ly-6G-FITC, MHC Class II- PE, F4/80-APC, CD4-FITC and CD8-PE BD Biosciences, San Jose, CA, multiple set of analyses were performed to accommodate different combination of markers with fluorochromes) and analyzed by flow cytometry using a BD FACSCanto. Live leukocytes were gated using forward scatter and side scatter parameters and analyzed using FlowJo software (TreeStar, Inc.). A. We identified four distinct populations as shown. B. Histogram showing F4/80+ cells from CD45(hi)CD11b(hi) cells. C. Histogram showing MHC class II+ cells, indicating microglial activation from CD45(int)CD11b(+). D. Dotplot showing ratio of CD4 and CD8 from CD45(hi)CD11b(−) and from CD45(hi)CD11b(+). (TIFF) [file pone.0016211.s004.tiff]
